# Immune Status Against Hepatitis B in Patients After Allogeneic Hematopoietic Cell Transplantation—Factors Affecting Early and Long-Lasting Maintenance of Protective Anti-HBs Titers

**DOI:** 10.3389/fimmu.2020.586523

**Published:** 2020-11-24

**Authors:** Agnieszka Piekarska, Piotr Wisniewski, Krzysztof Lewandowski, Lidia Gil, Piotr Trzonkowski, Maria Bieniaszewska, Jan Maciej Zaucha

**Affiliations:** ^1^ Department of Hematology and Transplantology, Medical University of Gdansk, Gdansk, Poland; ^2^ Department of Endocrinology and Internal Diseases, Medical University of Gdansk, Gdansk, Poland; ^3^ Department of Laboratory Medicine, Medical University of Gdansk, Gdansk, Poland; ^4^ Department of Hematology and Stem Cell Transplantation, Poznan University of Medical Sciences, Poznan, Poland; ^5^ Department of Clinical Immunology, Medical University of Gdansk, Gdansk, Poland

**Keywords:** hepatitis B vaccine, vaccination schedule, donor vaccination, hematopoietic cell transplantation (HCT), chronic graft versus host disease (GVHD)

## Abstract

The immunization of allogeneic hematopoietic cell transplantation (HCT) recipients against vaccine-preventable diseases is a part of posttransplantation guidelines. We conducted a prospective study to assess clinical and immunological parameters that would determine the response and long-term maintenance of protective antibody titers upon the hepatitis B virus (HBV) vaccination after HCT. The investigated variables included: vaccination of the HCT recipients and their donors prior to HCT, chronic graft versus host disease (cGVHD) and the timing of post-HCT vaccination, and B- and T-cell subtype status. Forty-two patients were immunized with three or more doses of recombinant hepatitis B surface antigen (rHBsAg) administered according to the individualized schedule of 0-1-2-6-(12) months. After vaccination, seroconversion was achieved in the whole group. The vaccines were categorized according to the antibody (Ab) titers as weak (WRs; 28.7%), good (GRs; 38%) or very good responders (VGRs; 3.3%). In multivariate logistic regression, severe cGVHD (OR= 15.5), and preceding donor immunization (OR= 0.13) were independent predictors of a weak response to vaccination. A prior belonging to the WR group impaired the durability of protection (OR= 0.17) at a median follow-up of 11.5 years. Patients with severe cGVHD showed a trend toward lower median Ab titers, although they required a higher rate of booster vaccine doses. All VGRs had CD4+ cells > 0.2 x 10^6^/L. There was a lower mean rate of CD4+IL2+ lymphocytes in WRs. Vaccination demonstrated the immunomodulatory effect on B-cell and T-cell subsets and a Th1/Th2 cytokine profile, while shifts depended on a history of severe cGVHD and the type of vaccine responder. To conclude, vaccination of HCT donors against HBV allows a better response to vaccination in the respective HCT recipients. Double doses of rHBsAg should be considered in patients with cGVHD and in those not immunized before HCT. A dedicated intensified vaccination schedule should be administered to WRs.

## Introduction

Allogeneic hematopoietic cell transplantation (HCT) is a curative cellular therapy for a variety of disorders (1). In posttransplantation care, a dysfunctional immune system and infectious complications pose serious problems (2–5). Moreover, an observed gradual loss of specific postvaccination immunity after HCT necessitates the immunization of HCT recipients against vaccine-preventable diseases (6, 7). Following HCT, hepatitis B virus (HBV) can trigger serious liver complications, including fulminant hepatitis. Repetitive exposure to medical procedures poses a risk of HBV transmission. Moreover, reverse seroconversion upon immunosuppressive treatment is reported in anti-HBc-positive patients in whom HBV infection was resolved before HCT (8, 9). Therefore, in countries with a high incidence of hepatitis B, immunization with recombinant hepatitis B surface antigen (rHBsAg) and the maintenance of protective anti-HBs antibodies (Abs) are especially justified (6, 10).

Due to deficiencies in humoral and cellular immunity, as well as altered mechanisms regulating immune reactions, the overall response rate (ORR) of transplant recipients is inferior compared to that of healthy people (11–15). However, for the sake of simplicity, a universal vaccination protocol for all transplant recipients does not consider differences in immune recovery in the distinct HCT platforms (16–21). Data on the durability of postvaccine protection in HCT recipients are limited. Long-lasting immunity depends on many variables, including the immunogenicity of vaccines, immunosuppressive treatment, and chronic graft versus host disease (cGVHD) (18, 19).

The clinical presentation of cGVHD mimics autoimmune diseases, and the organ-debilitating impact does not spare the immune system (22–26). Weak granulocyte chemotaxis, low response to mitogens, defects of the primary and secondary immune response to bacterial and polysaccharide antigens as well as functional hyposplenism are well-known phenomena (27, 28). A state of chronic inflammation, maintained by IL17, may lead to immune exhaustion, while dysregulated polyclonally activated lymphocytes do not properly recognize specific antigens (23, 29). Therefore, severe cGVHD itself could deteriorate the postvaccine immune responses and the maintenance of anti-HBV immunity.

We launched a prospective study aiming at identification of clinical and immunological factors that determine the response and long-term maintenance of protective antibody titers upon individualized vaccination with rHBsAg after HCT considering: donor/recipient serological anti-HBV status, incidence and severity of cGVHD, the timing of vaccination after HCT and the patient immune reconstitution. The serological monitoring included anti-HBs Ab levels tested before HCT, and after transplantation up to rHBsAg administration and postvaccination follow-up. Subpopulations of B-cell and T-cell compartments, as well as the cytokine Th1/Th2 profile, were evaluated in the perivaccination period. An additional goal of the study was to optimize a vaccination schedule and standardize posttransplantation anti-HBV surveillance.

## Material and Methods

### Patients

Criteria to initiate a vaccination program included a lack of previous vaccination after allogenic HCT, the remission of any underlying disease, the discontinuation of immunosuppressive treatment for at least 2 months before vaccination, a lack of active infection and signed informed consent. Patients with a history of cGVHD were accepted, provided that they did not suffer from an active disease requiring immunosuppressive therapy. Altogether, 62 Caucasian patients qualified, but for the homogeneity of the study group, patients allotransplanted with the use of reduced intensity/toxicity conditioning regimens were excluded from the final analysis.

Standard GVHD prophylaxis consisted of cyclosporine and a short course of methotrexate. Anti-thymocyte globulin administration (7.5 mg/kg) was a component of GVHD prophylaxis in HCT from matched unrelated donors (MUDs).

A final study group consisted of 48 patients who started a vaccination program between Dec 2003 and Mar 2006, including 42 individuals requiring vaccination with rHBsAg. The study was designed to compare the quality of the immune response between patients immunized in the early (between 6 and 24 months) and late periods (> 24 months) after transplantation.

### Vaccination Protocol

Vaccination against HBV was a part of the whole immunization schedule, consisting of vaccines against poliomyelitis (3 doses), tetanus (3 doses), diphtheria (3 doses), *Haemophilus influenzae* (2 doses), and HBV, administered simultaneously in separate parts of the body. Immunization against influenza was given seasonally once a year, while immunization against *Streptococcus pneumoniae* was performed with a 23-valent polysaccharide vaccine ≥ 12 months post-HCT, as conjugated vaccines were not available at that time (11).

The recombinant surface antigen of HBV gained from *Saccharomyces cerevisiae* and absorbed on aluminum compounds was used (Engerix B; GlaxoSmithKline Biologicals). The protocol consisted of 3 or more doses of the vaccine administered intramuscularly in 4- to 6-week intervals according to the following schedule: 0-1-2-6-(12) months. The first dose of the vaccine in every case was double (40 μg) the standard dose. The titer of anti-HBs Abs was checked 4 - 6 weeks after every dose, and the administration of subsequent doses depended on the grade of the response. A lack of seroconversion or a low titer of Abs (anti-HBs <10 mIU/ml) was followed by subsequent administration of a double vaccine dose until a titer of anti-HBs Abs >10 mIU/ml was achieved. In the case of seroconversion or an anti-HBs Ab titer >10 mIU/ml after the initial dose, the next doses were single doses (20 μg). After protocol completion, the anti-HBs Ab titer was monitored regularly during visits in the posttransplantation unit. Revaccination was prescribed in patients in whom protective immunity was lost, including patients with anti-HBc positivity.

Patients were divided into three types of responders, weak (WRs), good (GRs), and very good responders (VGRs), depending on the achieved titer of anti-HBs Abs, the administered vaccine doses, and the maintenance of protective levels of humoral anti-HBV immunity (**Table 1**).

**Table 1:** Criteria for the WR, GR, or VGR groups depending on the achieved anti-HBs titers, the quantity of injected doses, and the maintenance of high protective immunity.

Groups	Criteria
Weak responders (WRs)	- ≥ 4 doses to achieve anti-HBs Abs 10–100 mIU/ml or - anti-HBs Abs >100 mIU/ml achieved after 2–4 doses but maintained no longer than 1 year
Good responders (GRs)	- anti-HBs Abs >100 mIU/ml achieved after 3–4 doses and maintained at least 1 year
Very good responders (VGRs)	- anti-HBs Abs >100 mIU/ml achieved after 1–2 doses and maintained at least 1 year

### Chronic GVHD

Chronic GVHD diagnosis was based on data from patient medical records, and the criteria of NIH 2014 Consensus were retrospectively adopted (26).

### Flow Cytometry

Immunophenotyping was performed from heparinized peripheral blood according to standard procedures at least 3 times: before vaccination and during and after the completion of the basic vaccination protocol. Analyses were performed using triple-color flow cytometry (FC). All Abs used for immunofluorescent staining were obtained from Becton Dickinson (BD), and cells were acquired on a FACS Calibur (BD).

Cells were incubated with the following Ab-conjugates: γ1/γ2a-FITC/PE (clone X39/X40), CD3-PerCP (clone SK7), CD4-PE (clone SK3), CD4-PerCP (clone SK3), CD8-FITC (clone SK1), CD8-PerCP (clone SK1), CD45RA-FITC (clone L48), CD45RO-PE (clone UCHL-1), CD19-CyChrome (clone HIB19), CD27-PE (clone L-128), IgD-FITC (clone IA6-2), IgM-FITC (clone G20-127), IgG-FITC (clone G18-145), IFNγ-FITC (clone 25723.11), IFNγ-PE (clone 25723.11), IL2-FITC (clone 5344.111), IL2-PE (clone 5344.111), IL4-PE (clone 3010.211), IL5-PE (clone JES1.39D10), and IL10-PE (clone JES3.12G8).

Assessments of T-cell subsets were performed in whole blood, while for assessments of B-cell subsets and cytokine expression, peripheral blood mononuclear cells (PBMCs) were isolated. Cellular subpopulations were analyzed in the lymphocyte gate by positive signals above the isotype fluorescent control.

#### Isolation of PBMCs

PBMCs were separated by density gradient centrifugation on a Histopaq 1077 (Sigma). After washing in RPMI medium (Tominex) mixed with 10% FBS medium (Tominex), cellularity was assessed, and PBMCs were suspended in RPMI/FBS medium to achieve a lymphocyte concentration of 2x10^6^/ml. Some isolated lymphocytes were suspended in the medium for cell culture, and the rest were stained with Abs against B-cell markers.

#### Stimulation of T-lymphocytes with PMA and Ionomycin

Isolated lymphocytes were incubated with phorbol 12-myristate 13-acetate (PMA; Sigma-Aldrich) and ionomycin (Sigma-Aldrich) in the presence of an inhibitor of cytokine secretion, Brefeldin A (GolgiPlug; BD), in round-bottom 24-well plates for 5–6 h at 37°C in a 5% CO_2_ atmosphere according to a standard protocol from Laboratoire d’Immunologie CHU Rangueil (Toulouse, France), with modifications implemented by the first author (30, 31).

#### Staining of Surface Antigens and Intracellular Cytokine*s*

The staining of membrane antigens from whole blood was preceded by double lysis. Cell suspensions were incubated with cocktails of antibodies/conjugates for 30 min in darkness and then washed in PBS (5 min; RCF 650). After centrifugation, the supernatant was removed, and specimens were ready for acquisition. Samples requiring intracellular staining were fixed with 3% formaldehyde and permeabilized with Perm2 (BD). After 30 min of incubation in darkness with cytokine-targeting Abs/conjugates, cells were washed and suspended in 200 μl of 0.5% formaldehyde solution. Acquisitions with FC were performed within 24 h.

### Ethical Approval

This study was performed in accordance with the Declaration of Helsinki and received the approval of the Independent Bioethics Committee of the Medical University of Gdansk. Informed consent forms were signed by all participating patients, and the possible consequences of the study were fully explained.

### Statistical Analysis

Data analysis involved descriptive statistics, contingency tables, Pearson’s chi-square test and the Mann-Whitney-Wilcoxon U test. Logistic regression was used to examine the influence of selected predictors jointly. A p-value of less than 0.05 was considered signiﬁcant. All statistical analyses, data manipulation, and graphical plots were performed using the RStudio statistical software environment (version 1.1 with R.3.6.1).

## Results

### Patients’ Characteristics

The median ages at transplantation and vaccination were 35 and 39 years, respectively. Forty-two patients immunized with rHBsAg were divided into the early (38%) and late vaccination groups (62%) according to the time elapsed after HCT. **Table 2** presents detailed patient characteristics. Five patients with naturally acquired anti-HBV immunity and one patient with solely adoptive anti-HBV immunity transfer were included in serological monitoring (the so-called ‘initially nonvaccinated group’).

**Table 2:** Patient characteristics.

Age (at transplantation); median (range) years	35 (16–54)
Age (at vaccination); median (range) years	39 (19–57)
Sex: female/male	22/26
Primary disease: AML/ALL CML Other (MDS, PNH, CEL)	18 26 4
Chemotherapy preceding HCT: Yes/No	18/30
Conditioning regimen: TBICy/BuCy120	9/39
Type of donor: MUD/MSD	6/42
Source of hematopoietic cells: BM/PB	14/34
CD34+ cells dose (x10^6^/kg recipient body weight): BM median (range) PB median (range)	3.3 (1.2–5.4) 6.5 (2.7–8.9)
aGvHD grade 2-3 (%)	21 (43.8%)
cGVHD	30 (62.5%)
cGVHD no/mild/moderate/severe	18/9/10/11
CMV reactivations/median months post-HCT (range)	17 (35.4%)/4 (1–12)
Immunization with rHBsAg: total; early vs. late group	42; 16 (38%) vs. 26 (62%)

AML, Acute Myeloid Leukemia; ALL, Acute Lymphoblastic Leukemia; CML, Chronic Myeloid Leukemia; MDS, Myelodysplastic Syndrome; PNH, Paroxysmal Nocturnal Hemoglobinuria; CEL, Chronic Eosinophilic Leukemia; MUD, matched unrelated donor; MSD, matched sibling donor; BM, bone marrow; PB, peripheral blood; HCT, Hematopoietic Cell Transplantation; aGVHD, acute graft versus host disease; cGVHD, chronic graft versus host disease; CMV, cytomegalovirus; rHBsAg, recombinant hepatitis B surface antigen.

The evaluation of long-lasting anti-HBV immunity was possible in 43 patients. Five patients were excluded due to a short follow-up. The median follow-up in the analyzed vaccinated group was 11.5 (range, 5–16) years, while in the initially nonvaccinated group, it was 16.5 (range, 15–21) years.

### Immune Status Against HBV

#### Serological Status of Patients and Donors

Thirty (62.5%) patients were vaccinated with rHBsAg before HCT, with an ORR of 53%, including 6 patients achieving an anti-HBs Ab range of 10–100 mIU/ml and 10 patients with anti-HBs Ab levels >100 mIU/ml. Five patients with naturally acquired immunity were anti-HBc and anti-HBs Ab positive (>100 mIU/ml). In 20 (41.7%) patients with anti-HBs Abs < 10 mIU/ml, passive immunization with anti-HBs gamma-globulin was administered.

Twenty-five (58%) of 43 matched sibling donors (MSDs) were vaccinated before donation with an ORR of 60%, and 3 donors had protective anti-HBs Ab titers following HBV infection. In 19 (44.2%) HCTs from MSDs, both donors and recipients were vaccinated.

#### Maintenance of Anti-HBV Protection Post-HCT Before Vaccination

Three months, 6 months, and 1 year after HCT, anti-HBs Abs were detected in 87%, 69%, and 40% of patients, respectively (**Table 3**). Protective anti-HBs Ab levels > 10 mIU/ml were found 3 months, 6 months and 1 year after HCT in 42%, 33%, and 15% of patients, respectively.

**Table 3:** Maintenance of anti-HBV protection in the prevaccination and pre-revaccination period post-HCT.

Timepost-HCT	Detectable anti-HBs antibodies*	Anti-HBs 10 – 100 mIU/ml	Anti-HBs > 100mIU/ml
3 months	87%	42%	29%
6 months	69%	33%	12%
1 year	40%	15%	8%

*Including all patients with anti-HBs titer ≥ 0.5 mIU/ml.

Three months post-HCT, there were no significant differences in anti-HBV protection between those immunized actively and those immunized passively.

Six months post-HCT, in univariate analysis, anti-HBs Abs were detectable significantly more often in those vaccinated before HCT (p=0.025) or in the case of donor vaccination (p=0.006). In multivariate analysis, the odds of having anti-HBV protection (anti-HBs Abs levels > 10 mIU/ml) depended on a recipient vaccination prior to HCT (OR 8.9, 95% CI: 1.4 – 177.3; p= 0.052), or naturally acquired immunity (OR 13.1, 95% CI: 1.3–321.8; p= 0.048). Other analyzed predictors (passive immunization, donor immunization) were not significant in multivariate analysis.

**Figure 1:**
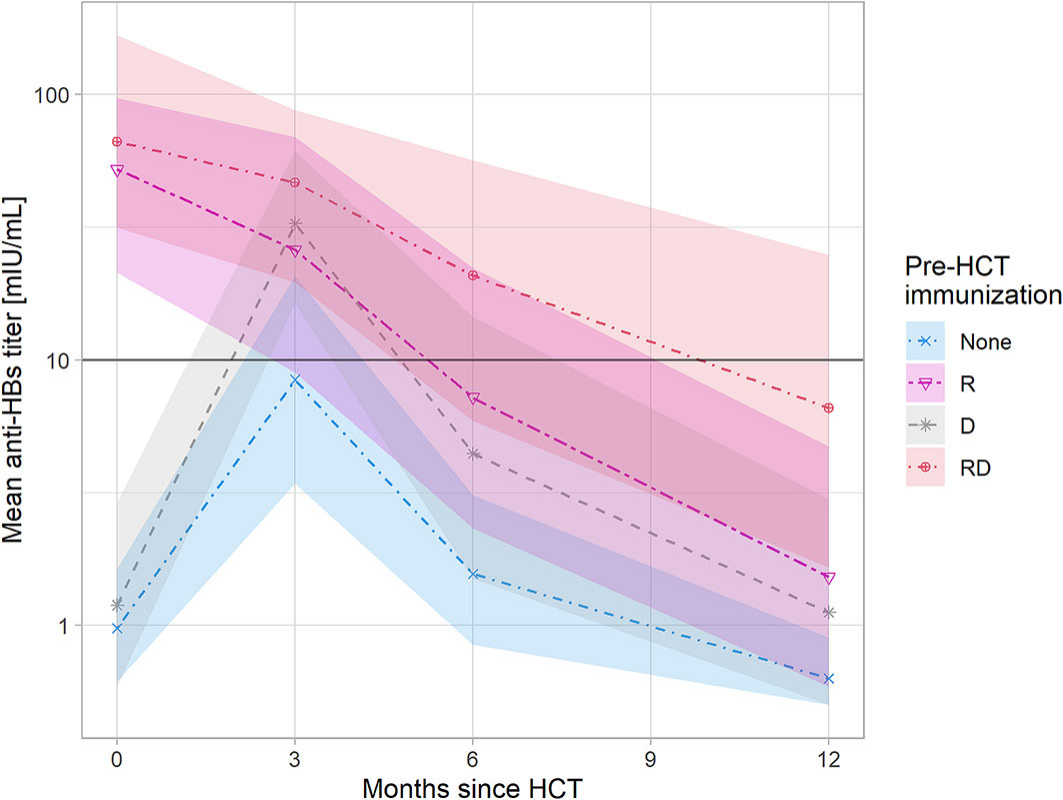
Anti-HBV protection up to one year post-HCT with respect to recipient and donor anti-HBV immunity. The shaded areas represent the 95% confidence intervals. Linear charts represent mean values of anti-HBs titers with respect to the anti-HBV immunity status of recipient and donor pre-HCT: - None - recipient nonimmunized actively or immunized ineffectively (anti-HBs<10 mIU/ml) and donor nonimmunized, - R - only recipient immunized, - D - only donor immunized, - RD - both recipient and donor immunized, A rise of anti-HBs titer 3 month post-HCT is caused by passive immunization with anti-HBs gamma-globulins prior to HCT administered to patients with anti-HBs<10 mIU/ml.

In univariate analysis, the maintenance of anti-HBV immunity at one year was significantly dependent on anti-HBs Ab levels > 10 mIU/ml in donors (p=0.006) and prior effective vaccination in the patients (p=0.04). In multivariate analysis, naturally acquired anti-HBV immunity (anti-HBc positivity, anti-HBs positivity) increased the odds of maintaining protective anti-HBs titers (OR 5.09, 95% CI: 0.9–32.0; p= 0.065). The remaining predictors (passive immunization, donor’s immunization) were not significant in multivariate analysis. The graphical presentation of anti-HBV protection up to one-year post-HCT with respect to recipient and donor anti-HBV immunity status is presented in **Figure 1**.

No reverse seroconversion was observed in the anti-HBc and anti-HBs Ab-positive patients. The adoptive transfer of anti-HBV immunity occurred in 10 patients with grafts from MSDs.

#### Response to Vaccination With rHBsAg: Patient-, Transplant-, and Donor-Related Factors

Seroconversion was achieved in the whole group. There were 12 (28.7%) patients classified as WRs, 16 (38%) as GRs, and 14 (33.3%) as VGRs. No severe complications related to the immunization of HCT recipients were recorded.

**Figure 2:**
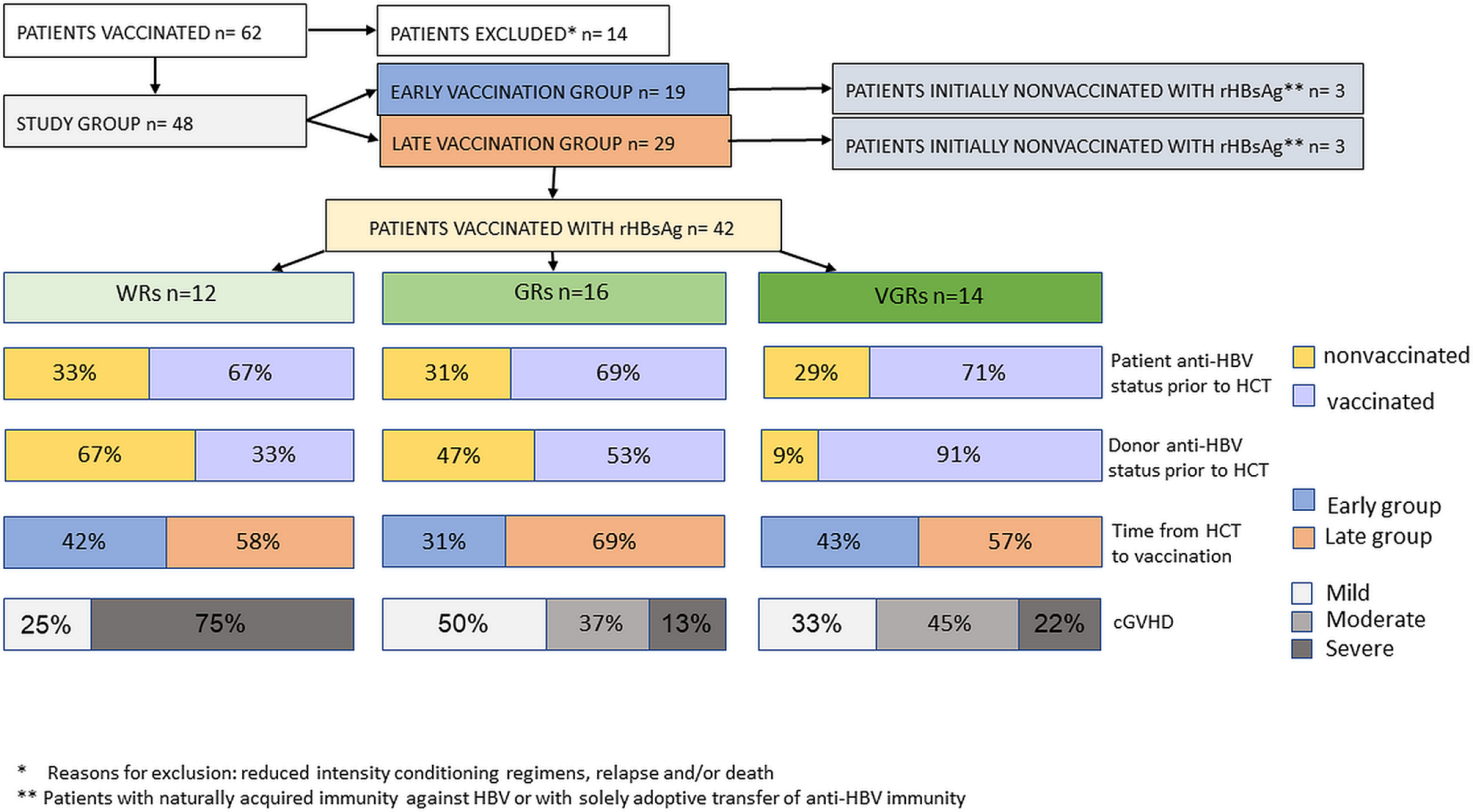
A structure of the study group and significant differences between the response groups with respect to patient-, transplant-, and donor-related factors.

Statistical analysis did not show the influence of various factors, including treatment with chemotherapy preceding HCT, the type of conditioning regimen, the source of hematopoietic cells, the type of donor, the patient’s previous vaccinations, reactivation of cytomegalovirus, of the patient’s age at transplantation and vaccination, on the results of active immunization after HCT in the study group. In univariate analysis, there was a trend toward the unfavorable impact of severe cGVHD on the results of active immunization with rHBsAg (p=0.057). Seroconversion after the first vaccine dose was significantly more frequent in patients who received transplants from donors immunized against HBV (p= 0.022). The majority (91%) of VGRs had immunized donors, in contrast to 33% of WRs (p=0.018). In 11 patients, adoptive immunity transfer was noted. Kaplan-Meier analysis indicated the positive impact of adoptive immunity transfer on postvaccination responses (p= 0.014). In the multivariate model, severe cGVHD increased the odds for WRs (OR= 15.5, 95% CI: 1.9–244.0; p= 0.02), while preceding donor immunization decreased the odds for WRs (OR= 0.13, 95% CI: 0.01–0.9; p= 0.05). A time of immunization ≥24 months after HCT (the late vaccination group) was inversely associated with weak response (OR= 0.43, 95% CI: 0.04–3.5; p= 0.4), but the effect did not reach statistical significance. Other analyzed predictors (sex, age >40, patient immunization before HCT) did not show an influence in multivariate analysis. The chart visualizing the study group and significant differences between the response groups is presented in **Figure 2**.

#### Evaluation of Long-Term Immunity

In the long-term study group anti-HBs Ab concentrations >100, 50–99, and 10–49 mIU/ml were detected in 30 (69.8%), 8 (18.6%), and 5 (11.6%) patients, respectively.

In the vaccinated group, the median anti-HBs Ab titer was 230 mIU/ml (range, 11 - >1,000 mIU/ml). Thirteen (35.1%) patients required reimmunization with booster doses of rHBsAg due to a decline in Abs. In 9 patients, one booster dose was sufficient, and 2 and 3 doses were administered in 3 and 1 patients, respectively. The majority (84.6%) of patients achieved anti-HBs Ab titers >100 mIU/ml upon reimmunization. There was no case of hepatitis B in the study group in the follow-up period.

In the initially nonvaccinated group, the median anti-HBs Ab titer was 561.5 mIU/ml (range, 91 - >1,000 mIU/ml). Two patients required one booster dose of rHBsAg due to a gradual loss of immunity to approximately 30 mIU/ml, and they achieved a long-term anti-HBs Ab titer > 100 mIU/ml upon reimmunization.

**Figure 3:**
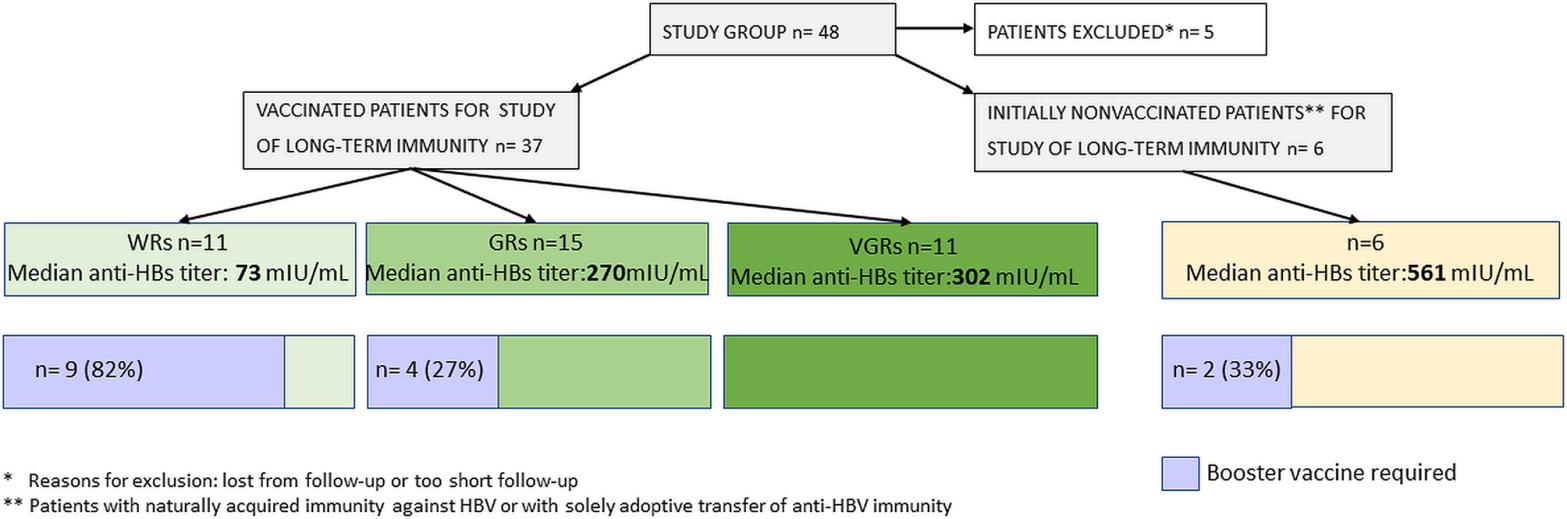
Differences in the long-term immunity between the response groups and a need for booster vaccine doses.

In univariate analysis, a prior belonging to the WR, GR, or VGR groups had a significant impact on median anti-HBs titers that were 73, 270, and 302 mIU/ml, respectively (p= 0.05). Booster rHBsAg doses were required in 9 WR patients and in 4 GRs but in no VGR patients (p< 0.0002). In multivariate analysis, the odds for maintaining anti-HBs titers > 100 mIU/ml were lower for the WR group (OR= 0.17, 95% CI: 0.02–1.02; p= 0.059). The chart visualizing the differences in the long-term immunity between the response groups and a need for booster vaccine doses is presented in **Figure 3**.

We observed higher median anti-HBs Ab concentrations in patients with previously noted adoptive immunity transfer (363.5 vs. 240 mIU/ml), and fewer patients required a booster dose of rHBsAg (20% vs. 56.6%), but statistical significance was not achieved.

The median long-term anti-HBs Ab concentrations did not differ significantly with the use of our vaccination protocol between patients vaccinated at age of < 40 and those vaccinated at age of ≥ 40 years (224.5 vs. 263 mIU/ml, respectively) in univariate and multivariate analyses.

We observed higher median anti-HBs Ab titers in patients without a history of cGVHD or with mild cGVHD (282.5 and 270 mIU/ml, respectively) than in patients with severe cGVHD (110 mIU/ml). In multivariate analysis, the odds for maintaining anti-HBs titers > 100 mIU/ml in patients with severe cGVHD were lower than those in patients with a mild form of or without cGVHD (OR= 0.4, 95% CI: 0.04 – 3.07; p= 0.41). Although the difference was not statistically significant, in 60% of the severe cGVHD patients, the Ab titers were raised by booster doses of rHBsAg in contrast to 32% of those with a mild form of or without cGVHD (p=0.09). The remaining analyzed predictors (sex, donor immunization, vaccination timing) did not show an influence in multivariate analysis.

### Immune Reconstitution

The adoptive T-cell and B-cell immunity recover within months or years in patients after HCT, and this process is very individual. Immune reconstitution depends on the pretransplant factors like an underlying disease and its treatment, age, conditioning regimen, donor, a source of hematopoietic cells, and posttransplant factors, e.g., GVHD. The recovery of B-cells is similar to ontogeny and usually quantitatively normalizes around 1 year post-HCT. However, a lowered cumulation of hypermutations in VH genes, impaired isotype switch and IgG production, processes dependent *inter alia* on Th2 cells, are frequently observed after HCT. In contrast, T-cell reconstitution is inverted, and memory/effector T-cells dominate even many years posttransplant, while the reconstitution of naïve T-cells, which broaden the repertoire of specificities, starts not earlier than 6 months post-HCT in the case of CD4+ cells (32).

The lowered ability to produce specific antibodies in response to vaccination, observed in a substantial proportion of HCT recipients, led to the creation of intensified vaccination schedules post-HCT, including vaccination against HBV. Upon injection, rHBsAg is lysed and processed by antigen-presenting specific B-cells and presented with MHC-II molecule to Th2 cells. Activated Th2 lymphocytes induce differentiation of B-cells to plasma cells, secreting HBsAg in high quantities to stimulate immune B-cell and T-cell memory (33). All types of immune cells involved in creating anti-HBV postvaccination immunity were included into analyses, but their reconstitution depends on the time elapsed from HCT to a great extent. Therefore, the parameters of immune reconstitution were analyzed with respect to timing from HCT and, subsequently, their impact was assessed with respect to response to vaccination with rHBsAg.

#### Comparison of the Early and Late Vaccination Groups

There were no significant differences concerning absolute lymphocyte count, gamma-globulin rate or IgG and IgM levels, while the median IgA concentration was significantly higher in patients > 2 years post-HCT (2.1 vs. 1.36 g/L; p= 0.005). The summarized comparison of detailed immune parameters described below is presented in **Table 4**.

**Table 4:** Detailed comparison of general characteristics and parameters describing immune reconstitution between the early vaccination group and the late vaccination group.

	Early group<24 months post-HCT	Late group>24 months post-HCT	Units	Statistics
General characteristics				
Female/male	42/58	47/53	%	p = NS
Age at vaccination*	**35** (21–50)	**43** (20–57)	years	**p = 0.002**
Time after HCT*	20 (12–24)	56 (31–119)	months	N/A
Time after immunosuppression taper*	7 (2–19)	20 (12–31)	months	N/A
Immunoglobulins				
IgG**	11.36 (± 4.11)	11.55 (± 2.56)	g/L	p = NS
IgA**	**1.36** (± 0.68)	**2.1** (± 0.97)	g/L	**p = 0.005**
IgM**	0.81 (± 0.42)	0.96 (± 0.45)	g/L	p = NS
Lymphocytes				
Absolute lymphocyte count**	2.05 (± 0.91)	2.42 (± 0.91)	x 10^9^/L	p = NS
B lymphocytes CD19+*	0.28	0.41	x 10^9^/L	p = NS
CD19+IgD(+)*	0.11	0.16	x 10^9^/L	p = NS
CD19+IgM(+)*	0.21	0.31	x 10^9^/L	p = NS
CD19+IgG(+)*	0.2	0.1	%	p = NS
Naïve CD19+IgM(+) CD27-*	50.0 0.14	42.6 0.17	% x 10^9^/L	p = NS p = NS
Naïve CD19+IgD(+) CD27-*	81.0 0.23	83.15 0.34	% x 10^9^/L	p = NS p = NS
Memory CD19+IgG(+) CD27+*	0.6	0.25	%	p = NS
Memory CD19+IgM(+) CD27+*	2.5 0.007	2.7 0.11	% x 10^9^/L	p = NS p = NS
Memory CD19+IgD(+) CD27+*	2.0 0.006	2.7 0.11	% x 10^9^/L	p = NS p = NS
Memory CD19+IgD(-) CD27(-)*	5.4 0.015	5.12 0.014	% x 10^9^/L	p = NS p = NS
T lymphocytes CD3+*	1.07	1.25	x 10^9^/L	p = NS
T lymphocytes CD3+CD4+*	**0.33**	**0.53**	x 10^9^/L	**p = 0.006**
T lymphocytes CD3+CD8+*	0.73	0.7	x 10^9^/L	p = NS
CD4/CD8 ratio*	**0.52**	**0.86**		**p = 0.008**
Naïve CD4+CD45RA+*	25.0 0.08	20.4 0.1	% x 10^9^/L	p = NS p =0.096
Memory CD4+CD45RO+*	61.0 **0.21**	65.9 **0.34**	% x 10^9^/L	p = NS **p = 0.011**
Naïve CD8+CD45RA+*	41.1 0.29	44.9 0.3	% x 10^9^/L	p = NS p = NS
Memory CD8+CD45RO+*	26.3 0.2	26.5 0.19	% x 10^9^/L	p = NS p = NS
Th1 cytokine expression*				
CD3+CD8-INFγ+	20.34	20.22	%	p = NS
CD3+CD8-IL2+	24.13	26.17	%	p = NS
CD3+CD8+INFγ+	**34.23**	**27.83**	%	**p=0.052**
CD3+CD8+IL2+	6.16	5.0	%	p = NS
CD3+ IL2- INFγ+	**49.14**	**38.13**	%	**p= 0.018**
CD3+ IL2+ INFγ-	**14.74**	**21.62**	%	**p=0.011**
Th2 cytokine expression*				
CD3+CD8-IL4+	1.79	2.15	%	p = NS
CD3+CD8-IL5+	0.09	0.15	%	p = NS
CD3+CD8-IL10+	0.12	0.12	%	p = NS
CD3+CD8+IL4+	0.41	0.48	%	p = NS
CD3+CD8+IL5+	0.05	0.04	%	p = NS
CD3+CD8+IL10+	0.04	0.03	%	p = NS

*Median value (range in parenthesis).

**Arithmetic mean value.

Bolded values showed statistical differences.

The reconstitution of B lymphocytes, including absolute counts of CD19+ cells and B-cell subpopulations with immunoglobulin IgD(+) and IgM(+) receptors, were comparable between the early and late group. The frequencies of CD19+IgG (+) cells were very low in both groups, which is why only percentages are provided. Naïve (CD27-) cells dominated over memory (CD27+) B-cells (p< 0.001), with a trend toward higher absolute values in the late group. The proportion and absolute counts of IgM(+)CD27(-) and IgD(+)CD27(-) naïve B-cells did not differ significantly between the two distinct groups, as was the case for IgM(+)CD27(+) and IgD(+)CD27(+) memory B-cells and double negative (DN) IgD(-)CD27(-) late differentiated memory B-cells.

The reconstitution of T lymphocytes did not vary concerning the proportion and absolute counts of CD3(+) and CD3(+)CD8(+) cells, while there were higher rates and absolute counts of CD3(+)CD4(+) cells in the late group: 18.2% (0.33 x 10^9^/L) vs. 25.6% (0.53 x 10^9^/L) (p= 0.006). The inverse CD4/CD8 ratio was more evident in the early group: 0.52 vs. 0.86 (p= 0.008). The naïve (CD45RA+CD45RO-) and memory (CD45RA-CD45RO+) subsets of CD8(+) cells were comparable, while naïve CD4(+) cells were less frequent than memory CD4(+) cells (p< 0.001). In the late group, there was a trend toward more numerous naïve CD4(+) cells (p=0.096), while memory CD4(+) cells achieved significantly higher values (p= 0.011).

The expression of cytokines from the Th1 profile (IFNγ+ and IL2+) dominated in both groups. IL2 predominated in CD3(+)CD8(-) cells while IFNγ predominated in CD3(+)CD8(+) cells with a trend to higher IFNγ expression in the early group (p=0.052). IL2(-)IFNγ(+) lymphocytes predominated in the early group (p= 0.018) and IL2(+)IFNγ(-) lymphocytes were prevalent in the late group (p=0.011). Within the Th2 cytokine profile, the expression of IL4 was higher in CD8(-) cells, and did not differ between the early and late groups. The Th1/Th2 ratio (IL2/IL4) was slightly higher in the early group: 11.94 vs. 9.62.

#### Variations in Parameters of Immune Reconstitution and Cytokine Profile Upon the Receipt of Vaccination

The whole VGR group achieved an absolute count of CD3(+)CD4(+) cells > 0.2 x 10^9^/L at vaccination. Among 6 patients who had not reached this value, half turned out to be GRs, and the remaining 3 were WRs. Insight into the naïve CD4(+) T-cell subset did not show significant differences in the immune response to vaccination for the cut-off of 0.05 x 10^9^/L, while there was a trend toward a lower RR for the cut-off of 0.03 x 10^9^/L, which was not reached in 63% of WRs (p= 0.057).

Generally, the levels of either naïve or memory T-cell compartments rose gradually during the realization of the vaccination protocol (p< 0.001). However, in the WR group, naïve CD4(+) cell counts began to rise with a delay compared to those in the GR and VGR groups (p< 0.001). We did not find a negative impact of a history of cGVHD on the number of naïve CD4(+) T-cell subsets.

In turn, the numbers of CD19(+) B-lymphocyte and naïve CD27(-) and memory CD27(+) subsets had no statistical impact on the immune response to rHBsAg. Nevertheless, the rates of memory IgM(+) and IgD(+)CD27(+) B-cell subsets showed a significant rise in the second measurement in GRs when the humoral response was observed (p=0.027 and p= 0.047, respectively). Interestingly, the median percentages of DN IgD-CD27- in WRs, GRs, and VGRs were 5.77%, 4.14%, and 5.44% within CD19+ B cells, respectively, and the difference was significant between WRs and GRs (p= 0.047). Concerning a history of cGVHD, the memory B-cell subsets IgM(+) and IgD(+) increased significantly upon vaccination in the second measurement only in patients with mild cGVHD (p<0.001), in contrast to patients with a moderate or severe form. We observed slightly lower median values of DN IgD-CD27- in patients without cGVHD and with a mild form of cGVHD 4.74% and 4.51%, respectively, while in patients with a history of moderate and severe cGVHD had median values 6.27% and 5.47% without statistical significance.

The rate of CD8(-) cells with IFNγ expression had no statistical impact on vaccination efficacy and did not change during vaccination. In contrast, the mean percentage of CD8(-)IL2(+) cells differed significantly between the WR group and VGR group (18.9 vs. 26.9%; p= 0.043) and decreased upon immunization in all patients. The mean values of CD8(-)IL2(+) cells decreased upon vaccination in patients without cGVHD and with a mild form of cGVHD (23.8 and 20.1% vs. 16.1 and 14.7%, respectively), while these values remained at a stable level of approximately 20% in patients with a history of severe cGVHD. The rates of IL4-expressing cells increased gradually in the WR group upon vaccination in subsequent analyses (2.3 vs. 2.9 vs. 3.2%; p= 0.033). In patients with severe cGVHD, a similar increase in the IL4(+) cell rate was observed during vaccination (2.6 vs. 3.3 vs. 3.5%; p= 0.012, p= 0.024). The rate of IL5(+) T lymphocytes rose already in the second measurement in GRs (p= 0.023) and VGRs (p<0.001), and a delayed rise after vaccination completion was observed in WRs (p= 0.01). In patients with mild cGVHD or without a history of cGVHD, the IL5(+) cell rate increased in the second measurement (p= 0.027 and p<0.001, respectively), whereas a delayed rise was present in patients with moderate and severe cGVHD (p= 0.017 and p= 0.038, respectively).

## Discussion

Despite the high immunogenicity of the anti-HBV vaccine, approximately 5% of healthy vaccinees fail to mount an adequate humoral response. The response rate in immunocompromised patients is reported to be lower. Preceding therapy, a vaccination schedule, and a dose of antigen are postulated to have a significant impact on immune responses (34). The immunogenicity of the primary vaccination is known to last 10 to 31 years, but not in the case of immunocompromised patients (34). In the analysis performed by Kaloyannidis et al., the probability of losing HBV immunity was 100% at 5 years post-HCT for patients who received transplants from nonimmunized donors and 78% and 58% for those who received transplants from vaccinated donors and naturally immunized donors, respectively (6). In contrast to our study group, those patients were not revaccinated post-HCT, and those data cannot be directly compared to our results. The aforementioned study by Kaloyannidis et al. also reported a high probability of reversed seroconversion, reaching 18% at 12 years (6). In the analysis by Mikulska et al., HBV reactivations were observed in 10% of patients with a median time of 19 months post-HCT (8). In our study group, from 5 patients with naturally acquired immunity, no reverse seroconversion was observed. Two patients received booster doses of rHBsAg as prevention when a gradual drop in anti-HBs Abs was observed.

A detailed analysis of our data from the early posttransplant period showed an evident impact of either patients’ or their donors’ vaccination status before HCT. In the case of insufficient or no protection against HBV, passive immunization remains the only solution. However, passively transferred anti-HBs Abs have a limited lifespan, as shown in our study. Therefore, repetitive infusions would be required to preserve sufficient protection. More prolonged protective anti-HBs titers were observed in patients effectively vaccinated before HCT and in those who received transplants from vaccinated donors. Moreover, donor immunization provides an additional benefit for an HCT recipient - memory B and T-cells responsible for the adoptive transfer of immunity, which can be easily recalled by a booster dose of vaccine (34, 35).

The ORR to post-HCT vaccination of 100% and the long-lasting maintenance of anti-HBV immunity might be astonishing given that these vaccinations were administered to immunocompromised patients. Similar results were reported by Machado et al. in 45 recipients immunized ≥ 1 year after allo-HCT, while in another cited study, the seroconversion rate in 168 adult patients was 59% (13, 36). We used three known strategies to improve the immune response: an increased vaccine dose to 40 μg administered until seroconversion was achieved, an intensified dosing schedule and the co-administration of rHBsAg with other vaccines from the vaccination protocol (34). A higher dose of the vaccine administered initially augments the B-cell response and increases the proportion of memory B-cells, which could also have an impact on the duration of long-lasting immunity (37). Moreover, in the study group, more than 90% of the VGRs received transplants from vaccinated donors, thereby transferring memory cells to their recipients. The positive effect of prior donor immunization was also confirmed in the multivariate analysis.

The median follow-up in our study, exceeding 10 years, enabled the monitoring of a long-lasting anti-HBV immune status in the majority of patients. The inevitable loss of anti-HBV immunity was confirmed in HCT recipients, but the rate was dependent on the degree of the immune response to the primary inoculum series (37). No patient from the VGR group required any booster dose of rHBsAg, and only 20% of patients with previously noted adoptive immunity transfer required a booster dose. In contrast, primary WRs, according to our predefined criteria, should be closely monitored as booster doses of rHBsAg are likely to be needed. However, one can expect an intense reaction of memory cells to recall antigen, as in most cases, one booster dose was sufficient to achieve anti-HBs Abs titers > 100 mIU/ml.

Upon injection, rHBsAg is lysed and processed by antigen-presenting specific B-cells and presented with MHC-II molecule to Th2 cells. Activated Th2 lymphocytes induce differentiation of B-cells to plasma cells, secreting HBsAg in high quantities to stimulate immune B-cell and T-cell memory (33). To better understand the immune response in HCT recipients, we measured several parameters of the immune system, including subpopulations of B and T-cells, with deep analysis of subsets expressing Th1 and Th2 cytokine profiles. We confirmed that it is optimal to start active immunization when the absolute CD4(+) T cell count exceeds 0.2 x 10^9^/L. Similar to HCT recipients, in patients with HIV infection, the seroconversion rate after HBV vaccination was shown to be directly proportional to the CD4(+) cell count (34). The repertoire of naïve T lymphocytes is crucial for the optimal response to antigens and depends on thymus regeneration starting from 6 to 12 months posttransplantation (38). In our study group, there was a trend toward a weaker response in those with naïve CD4(+) cells below 0.03 x 10^9^/L, which was already reported by Roux et al. in HCT recipients vaccinated with tetanus toxoid (14). We did not observe a decreased proportion of Th2-like cytokine-producing CD4+ cells, while IL2-producing CD4+ cells were significantly lower in WRs, as described in “*in vitro*” studies (39). Moreover, the realization of an active immunization protocol led to a gradual increase in IL4(+) and IL5(+) cells and a decrease in IL2(+)CD4+ lymphocytes.

The early vaccination group had significantly lower IgA immunoglobulin levels than the late group, which could reflect an impaired isotype switch from naïve to memory B-cells. However, we did not confirm the influence of median IgA levels on the quality of the postvaccination humoral response (15, 16). Differences in circulating B-cells at the time of vaccination did not show significant impact on the immune response (15). However, the humoral response was parallel with the increase in memory IgM(+) and IgD(+) B-cell subsets. Interestingly, we found a significantly higher percentage of late differentiated memory DN B lymphocytes in WRs. This subpopulation is reported to reflect senescence of the immune system related to chronic inflammatory processes, e.g., HIV infection, lupus, Alzheimer’s disease (40). This observation could be explained by a high rate of patients with a history of severe cGVHD in the WR group.

The comparison of the early and late groups showed some differences in immune reconstitution but these parameters had ultimately no impact on the quality of the post-vaccination response in our study group neither in the univariate nor multivariate analysis. In multivariate analysis, there was only a slight trend toward less common weak responses to immunization with rHBsAg in the late vaccination group. This is another suggestion that vaccination against hepatitis B started at 6 months post-HCT according to the international recommendations is absolutely rational.

Severe GVHD induces structural damage that has a serious and durable impact on thymus functioning and output (41–43). A direct influence of immunosuppressive agents in our study group was excluded since immunosuppression was discontinued at least 2 months before enrollment. However, a history of severe cGVHD significantly influenced the response to anti-HBV vaccination and the maintenance of protective immunity, even though immunization was performed in the nonactive phase of the disease. We also noted a delayed increase in Th2 IL4(+) and IL5(+) cells in response to rHBsAg in WRs and patients with a history of severe cGVHD. Our observations are in line with those of the study by Jaffe et al., in which the seroconversion after 3 doses of rHBsAg was observed in 64% of patients after HCT, and the negative influence of a GVHD history was also demonstrated (13). Furthermore, Kaloyannidis et al. indicated cGVHD as an independent factor for anti-HBs Ab disappearance (6).

Guidelines for the prevention of infectious complications among HCT recipients recommend the same vaccination schedule for all HCT recipients, and active immunization of patients with cGVHD should not be postponed (18, 20, 21, 36, 38). Evidence exists that severe cGVHD and its treatment deteriorate the efficacy of active immunization (14, 18). Therefore, immunization should not begin during the exacerbation of GvHD and the escalation of immunosuppressive treatment. Instead, effective anti-infectious prophylaxis should be provided (18).

Our study has several limitations. First, a laboratory part was designed and performed when the serological assessment of antibody response was only available for vaccination against HBV in the hospital laboratory. Therefore, we did not analyze the response rates to the remaining co-administered vaccines. Second, although we did not observe any case of hepatitis B in our study group, which gives evidence for clinical protection of patients with serological response to vaccination, we did not investigate the specific cellular immunity. The modern assays such as Ag-specific cell detection, protein quantification, and transcriptomics techniques would give a deeper insight into post-vaccine immunity and potential correlation between the humoral and cellular anti-viral protection. Third, immunophenotyping was performed with the use of 3-color flow cytometry as at the time of laboratory analyses it was the only available equipment. The multicolor flow cytometry available nowadays would extend the analytic possibilities.

In summary, vaccination of recipients and their donors against HBV prior to HCT is beneficial in many aspects, including protection in the early posttransplantation period. Our results add important information that might help the clinical management of HCT recipients by implementing a double dose of rHBsAg (40 μg) in patients with a history of cGVHD and those not immunized before HCT or those who received transplants from nonimmunized donors until seroconversion is achieved. The subsequent doses might be reduced to the standard 20 μg. Second, an intensified vaccination schedule of 0-1-2-6-(12) months is advised for WRs who do not achieve a protective anti-HBs Ab titer >10 mIU/ml or in those for whom the level is between 10 and 100 mIU/ml after 3 doses of HBV vaccine. Third, a titer of anti-HBs Abs should be monitored routinely in WRs and patients with cGVHD, since a decline in specific Abs requiring reimmunization is expected. Finally, in GRs and VGRs and patients without cGVHD, the titer of anti-HBs Abs can be measured in longer (e.g., 5-year) intervals.

## Data Availability Statement

The original contributions presented in the study are included in the article/supplementary materials. Further inquiries can be directed to the corresponding author/s.

## Ethics Statement

The studies involving human participants were reviewed and approved by Independent Bioethics Committee of the Medical University of Gdansk. The patients/participants provided their written informed consent to participate in this study.

## Author Contributions

AP was involved in the conception and design of the study, the acquisition of data, statistical analysis, and the analysis and interpretation of data, and writing of the manuscript. PW took part in the statistical analysis and critical revision. LG was involved in the data analysis and critical revision. KL took part in the data analysis, drafting of the article, and critical revision. PT was involved in the data analysis and critical revision. MB took part in the data analysis and critical revision. JZ was involved in the data analysis, drafting of the article, and critical review. All authors contributed to the article and approved the submitted version.

## Funding

The laboratory analyses (immunophenotype of lymphocyte subsets and cytokine expression) were financed by a grant from the Ministry of Science and Higher Education (Mentorship Grant number 2 PO5B 080 26). This work was supported by the French Government and the French Embassy in Poland (science.varsovie-amba@diplomatie.gouv.fr). The competence to assess Th1 and Th2 cytokine expression was gained thanks to the French Government Scholarship (Number 385816B/P361471J) taking place in Laboratoire d’Immunology CHU Rangueil, Toulouse, France, in 2003.

## Conflict of Interest

The authors declare that the research was conducted in the absence of any commercial or financial relationships that could be construed as a potential conflict of interest.
